# Transcriptional Regulation of Protein Synthesis by Mediator Kinase Represents a Therapeutic Vulnerability in MYC-driven Medulloblastoma

**DOI:** 10.21203/rs.3.rs-5329081/v1

**Published:** 2024-11-01

**Authors:** Dong Wang, Caitlin Ritz, Angela Pierce, Bethany Veo, Yuhuan Luo, Breauna Brunt, Nathan Dahl, Ammu Suresh, Natalie Serkova, Sujatha Venkataraman, Etienne Danis, Kamil Kuś, Milena Mazan, Tomasz Rzymski, Rajeev Vibhakar

**Affiliations:** 1 Department of Pediatrics, University of Colorado Anschutz Medical Campus; Aurora, CO, USA; 2 Morgan Adams Foundation Pediatric Brain Tumor Research Program, Children’s Hospital Colorado; Aurora, CO, USA; 3 Department of Neurosurgery, University of Colorado Anschutz Medical Campus; Aurora, CO, USA; 4 Department of Surgery, University of Colorado Anschutz Medical Campus; Aurora, CO, USA; 5 Department of Radiology, University of Colorado Anschutz Medical Campus; Aurora, CO, USA; 6 Biostatistics and Bioinformatics Shared Resource, University of Colorado Cancer Center, Aurora; CO, USA; 7 RVYU Therapeutics; Krakow, Poland

## Abstract

MYC-driven medulloblastoma (MB) is a highly aggressive cancer type with poor prognosis and limited treatment options. Through CRISPR-Cas9 screening of MB cell lines, we identified the Mediator-associated kinase CDK8 as a critical regulator of MYC-driven MB. Loss of CDK8 substantially reduces MYC expression, induces pronounced transcriptional changes, suppresses monosome assembly, and decreases ribosome biogenesis and protein synthesis, consequently inhibiting MB growth. Mechanistically, CDK8 regulates the occupancy of RNA polymerase II at specific chromatin loci, facilitating an epigenetic alteration that promotes the transcriptional regulation of ribosomal genes. Targeting CDK8 effectively diminishes the stem-like neoplastic cells characterized by hyperactive ribosome biogenesis. Furthermore, we demonstrated that the combined inhibition of CDK8 and mTOR synergizes to optimize therapeutic outcomes *in vivo* and *in vivo*. Overall, our findings establish a connection between CDK8-mediated transcriptional regulation and mRNA translation, suggesting a promising new therapeutic approach that targets the protein synthesis for MYC-driven MB.

## Introduction

Medulloblastoma (MB) is the most common malignant pediatric tumor, accounting for 15–20% of childhood brain tumors^1^. Molecular profiling and genetic analysis categorized MB into four subgroups: WNT, SHH, Group 3, and Group 4^2,3^. Among these groups, patients with MYC-driven Group 3 MB (G3-MB) commonly experience relapse accompanied by metastatic spread and local recurrence, resulting in long-term survival rates of less than 5%^4^. To date, targeted options for G3-MB tumors are lacking, in part because of the incomplete understanding of tumorigenic mechanisms.

Dysregulated expression of the MYC proto-oncogene contributes to the development of many types of human cancer^5^. Numerous studies have demonstrated that MYC plays a pivotal role in regulating protein synthesis^6–9^. MYC affects cell proliferation, growth, and nucleolar size, and is associated with marked changes in the total rate of protein synthesis^10^. It also regulates ribosome biogenesis either directly by upregulating ribosomal RNA and protein components through chromatin structure remodeling, or indirectly by controlling essential auxiliary factors involved in rRNA processing, ribosome assembly, and subunit transportation from the nucleus to the cytoplasm^6,8,11–13^. Chromatin remodeling is an essential aspect of these processes through which MYC directly activates RNA polymerases^14–16^. Understanding dysregulated protein synthesis in MYC-driven oncogenesis is crucial for developing targeted therapeutic interventions that leverage the inherent vulnerabilities of these pathways in the context of tumor development.

The Mediator Kinase cyclin-dependent kinase 8 (CDK8) associates with the mediator complex, a large multi-subunit complex that regulates transcription by connecting enhancer-bound transcription factors to RNA polymerase II^17,18^. Overexpression of CDK8 has been demonstrated in various types of cancer, including colon cancer, breast cancer, glioblastoma, and hepatocellular carcinoma, making it a potential therapeutic target^19–22^. Several studies have determined the efficacy of CDK8 inhibitors in preclinical cancer models^23–27^. Importantly, unlike other transcriptional CDKs, CDK8 is not essential for basal transcription; instead, it plays a key role in driving transcriptional responses to stress responses and developmental stimuli^28–30^.

In this study, we found that CDK8 is an essential gene for MB growth. Importantly, MYC-driven medulloblastoma exhibited the most significant susceptibility to the loss of CDK8 among all cancer types. CDK8 depletion suppressed protein synthesis, suggesting that it cooperates with MYC to drive tumorigenesis. Mechanistically, we demonstrated that the loss of CDK8 induces pronounced transcriptional changes, resulting in the suppression of ribosomal gene expression, and impeding the growth of MYC-driven MB. Furthermore, CDK8 inhibition with a novel inhibitor, RVU120, synergizes with mTOR inhibition to suppress MYC-driven MB. This work holds the promise of significantly advancing our understanding of MYC-driven oncogenesis and provides critical preclinical data essential for the development of novel therapies targeting CDK8 and mTOR in MYC-driven medulloblastoma.

## Results

### CDK8 is a specific vulnerability in MYC-driven medulloblastoma

**Fig. 1:**
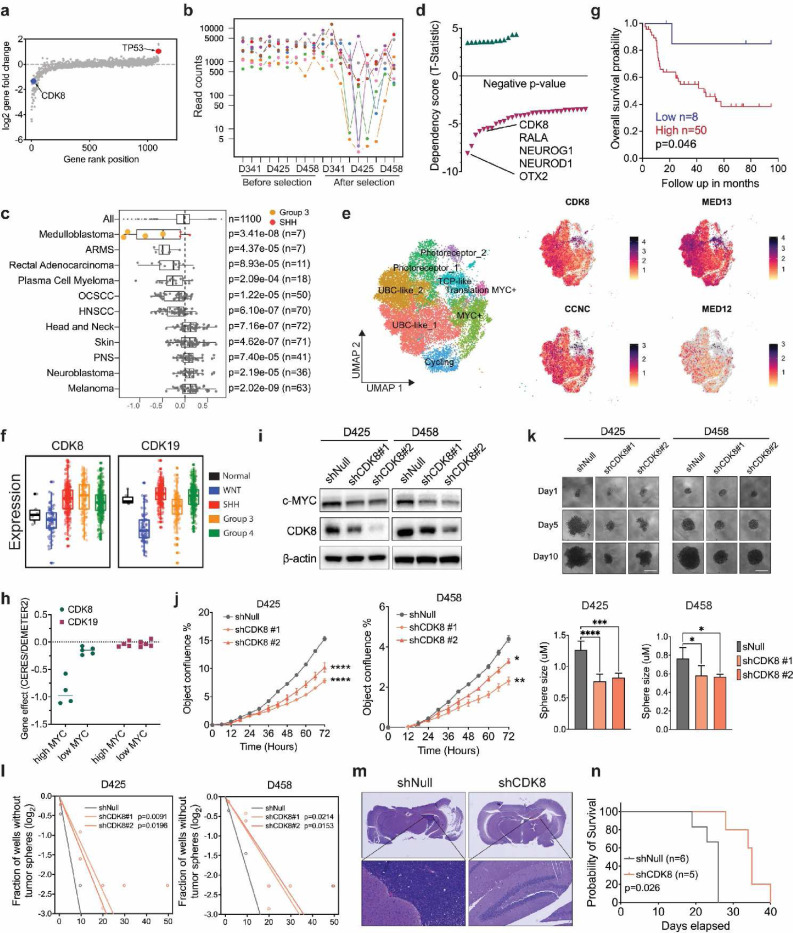
CDK8 is a specific vulnerability in MYC-driven medulloblastoma. **a.** Log-fold change of gene expression in CRISPR-Cas9 screening across D425, D458, and D341 MB cell lines. **b.** The read counts of sgRNAs of CDK8. Each dot represents a sgRNA targeting CDK8. **c.** The enriched lineages plot from DepMap indicates the DEMETER2 score of CDK8 across cancer types. n indicates the number of cell lines plotted in that lineage. **d.** The Dependency score from DepMap specific for MB. Labels below zero denote genes that are essential for MB growth. **e.** scRNA-Seq analysis of GP3 cells from seven patient samples. **f.** Microarray analysis of CDK8 and CDK19 expression in four subgroups of 763 MB patient samples. n = 6 normal samples were collected by our institution. **g.** Kaplan-Meier analysis demonstrating the association between CDK8 and survival within the MYC-high patient cases. **h.** The DEMETER2 scores of CDK8 and CDK19 in high MYC and low MYC MB cell lines. **i.** Immunoblot demonstrating the protein level of CDK8 in MB cells with the loss of CDK8. **j.** Growth of shNull and shCDK8-transduced D425 or D458 cells assayed in Incucyte live-cell analysis system. n = 5. Mean ± SD. Statistical analysis: one-way ANOVA. **k.** Images of neurosphere size in CDK8 knockdown and control cells are shown. Scale bar, 400 μm. Barplot is for Day 10. **l.** Sphere formation efficiency and self-renewal capacity were measured using extreme in vitro limiting dilution assays (ELDA) in shNull or shCDK8 transfected MB cell lines. P values were determined using the likelihood ratio test. **m.** Images showing a reduction in tumor formation of shCDK8 xenograft. Three mice from each group were sacrificed 20 days after tumor implantation. **n.** Kaplan-Meier analysis of D458 xenograft mice injected with shNull or shCDK8 cells.

To systematically identify genes representing therapeutic vulnerabilities in MYC-driven MB, we performed CRISPR-Cas9 screening targeting of 1140 druggable genes across three MYC-amplified human G3-MB cell lines^31,32^. CDK8 was identified as an essential gene for MB tumor growth (Fig. 1a,b and Fig S1a). We next explored the significance of CDK8 by leveraging the Cancer Dependency Map (DepMap), a platform that utilizes gene knockout or knockdown to map gene dependencies across hundreds of cancer types^33^. CDK8 is critical for various types of cancer, with MYC-driven medulloblastoma being the most sensitive cancer type to the loss of CDK8 (Fig. 1c). CDK8 stands out as a top dependency, similar to OTX2, Neurog1, and Neurod1, well-established genes that sustain stemness and drive proliferation in medulloblastoma (Fig. 1d)^34–37^. Furthermore, CDK8 is the only gene with clinically relevant inhibitors among the top dependencies, making it a potential target for the treatment of G3-MB.

Single-cell murine cerebellar transcript analysis showed relatively low expression of CDK8 and the Mediator Complex genes in normal tissues (Fig. S1b). To examine CDK8 in MB, we analyzed single-cell RNA-seq from seven patient samples and identified eight clusters within a total of 12,595 GP3 neoplastic cells. Genes associated with the Mediator Complex were expressed across all transcriptionally distinct cell clusters (Fig. 1e). Using a cohort of 763 described MB samples along with normal cerebellar samples, we found that G3-MB expressed higher levels of CDK8 than the normal cerebellum, particularly in subtypes Group 3β and 3γ with overexpressed c-MYC (Fig. 1f and Fig. S1c). In contrast, its paralog CDK19 was not overexpressed in Group 3 MB. Kaplan–Meier survival analysis performed on the same dataset revealed a correlation between CDK8 expression and poor overall survival in high-MYC MB (Fig. 1g). Consistent with these findings, CDK8 exhibited notably higher protein levels in multiple G3-MB cells than in the normal cerebellar tissue (Fig. S1d). Interestingly, the dependency on CDK8 was observed only in high MYC MB, but not in low MYC MB, suggesting its role in collaboration with MYC to regulate the transcriptional program (Fig. 1h).

To determine the dependency of MYC-driven MB on CDK8, we inhibited CDK8 expression in MB cells using lentivirus-mediated CDK8 shRNA. Loss of CDK8 led to a notable decrease in both MYC levels and cell proliferation (Fig. 1i,j). CDK8 depletion also significantly decreased neurosphere growth in MB cells (Fig. 1k). Extreme limiting dilution analysis showed that CDK8 depletion diminished self-renewal capacity and neurosphere formation efficacy, suggesting a role of CDK8 in mediating stemness and differentiation in G3-MB (Fig. 1l). To further examine the *in vivo* effects of CDK8 on tumor formation, MB cells transduced with either a control shRNA sequence (shNull) or shRNAs targeting CDK8 (shCDK8) were implanted intracranially into immunodeficient mice. Knockdown of CDK8 inhibited tumor growth and prolonged the survival of intracranial tumor-bearing mice relative to shNull, reinforcing CDK8 as a crucial factor controlling the growth of MYC-amplified MB (Fig. 1m,n and Fig. S1e).

### RVU120 suppresses the growth of medulloblastoma cells

**Fig. 2:**
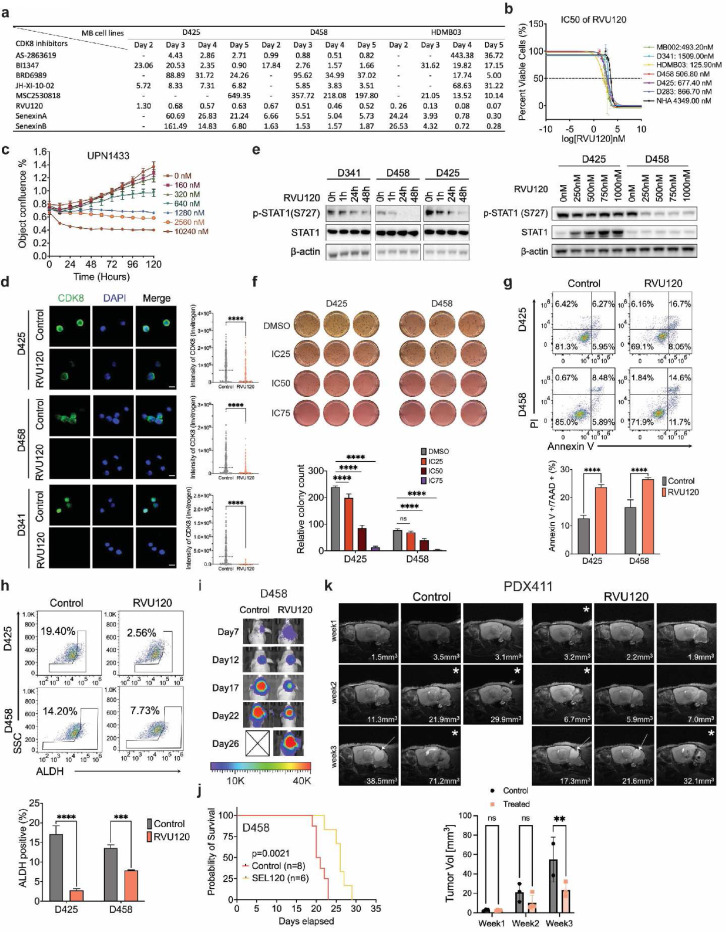
RVU120 suppresses the growth of medulloblastoma cells. **a.** IC50 determination of various CDK8 inhibitors in MB cell lines. Unit: μmol. **b.** IC50 of RVU120 at 72 h in MB cell lines and NHA cells. **c.** Dose-dependent proliferation curve of RVU120 treated primary MB cells from a G3-MB patient. **d.** Immunofluorescence of CDK8 (green) and DAPI (blue). MB cells were treated with IC50 RVU120 for 48 h. Scale bar, 10 μm. **e.** Immunoblot demonstrates the p-STAT1(S727) protein level with treatment of RVU120 across MB cell lines. **f.** Methylcellulose assay in MB cells treated with RVU120. **g.** Annexin V apoptosis assay. MB cells were treated with IC50 RVU120 for 48 h. **h.** Identification of the brain tumor-initiating cell fraction in MB cells by ALDH expression demonstrates a decrease in the ALDH^+^ fraction following IC50 RVU120 treatment for 48 hours. **i.** Representative bioluminescence images of mice treated with RVU120 (40mg/kg, daily, oral gavage) compared with vehicle. **j.** Kaplan–Meier survival curves for animals treated with control or RVU120. **k.** Representative axial T2-weighted turboRARE MRI sequences of mice treated with RVU120 or vehicle. PDX411 xenograft mice were treated with RVU120 (40 mg/kg) for 14 days, starting with the first MRI scan. The asterisks indicating that the mice exhibited a spongy tissue texture. An adjusted texture analysis was performed to measure the tumor size. One control mice died before the final scan.

We examined the localization of CDK8 using two CDK8 antibodies across various MB cell lines, human astrocytes (NHA), and a mouse embryonic fibroblast cell line (NIH3T3). Immunofluorescence analysis revealed predominant CDK8 expression within the nucleus, accompanied by additional expression in the cytoplasm (Fig. S2a). Several small-molecule inhibitors targeting CDK8 are currently undergoing preclinical development^24–27^. Our evaluation of eight CDK8 selective inhibitors demonstrated a broad range of half-maximal inhibitory concentrations (IC50) across three G3-MB cell lines. Among these, RVU120 exhibited remarkable potency, with the lowest IC50 values (Fig. 2a and Fig. S2b). We assessed the IC50 of RVU120 in various MB and NHA cell lines. In G3-MB cells, the 72-hour IC50 concentration ranged from 125.90 1509.00 nM. NHA displayed significantly higher resistance to RVU120, with an IC50 concentration of 4349.00 nM (Fig. 2b). Importantly, RVU120 treatment reduced the viability of patient-derived primary G3-MB cells, further confirming the efficacy of RVU120 in treating G3-MB (Fig. 2c).

Treatment with RVU120 led to decreased CDK8 expression and a concurrent reduction in p-STAT1 levels, which is a direct target of CDK8^38^ (Fig. 2d,e and Fig. S3a). Using a methylcellulose colony-forming assay and live cell imaging, we found that CDK8 inhibition suppressed colony formation and neurosphere growth in G3-MB cells (Fig. 2f and Fig. S3b, c). Additionally, flow cytometry analysis revealed a substantial increase in the total percentage of apoptotic cells following RVU120 treatment, as determined by both annexin V and active caspase 3 staining using flow cytometry (Fig. 2g and Fig. S3d). RVU120 treatment led to a reduction in neurosphere formation efficacy and the ALDH^+^ cell population, indicative of a decrease in the brain tumor-initiating cell fraction within a given cell population associated with stem-like properties such as self-renewal (Fig. 2h and Fig. S3e). A similar effect was observed with another CDK8-selective inhibitor, BI1347 (Fig. S3f, g).

To assess the potential intracranial efficacy of RVU120 *in vivo*, we evaluated its unbound partition coefficient, which determines the concentration of the compound in the CSF, corresponding to its free concentration in the brain. A ratio value of approximately 0.4 was observed, indicating permeation into the brain^39^ (Fig. S3h). Furthermore, in the D458 injected MB xenograft model, we found that administration of RVU120 extended the survival of mice in the treatment group (Fig. 2i,j and Fig. S3i). In a patient-derived xenograft G3-MB model (PDX411), three-dimensional volumetric analysis of T2-turboRARE MRI sequences showed a significant decrease in tumor size after 14 days of RVU120 treatment compared with the control (Fig. 2k). Collectively, these findings reveal an oncogenic role of CDK8 in MB and highlight the therapeutic potential of RVU120 for treatment of G3-MB.

### CDK8 depletion leads to repression of protein synthesis

**Fig. 3:**
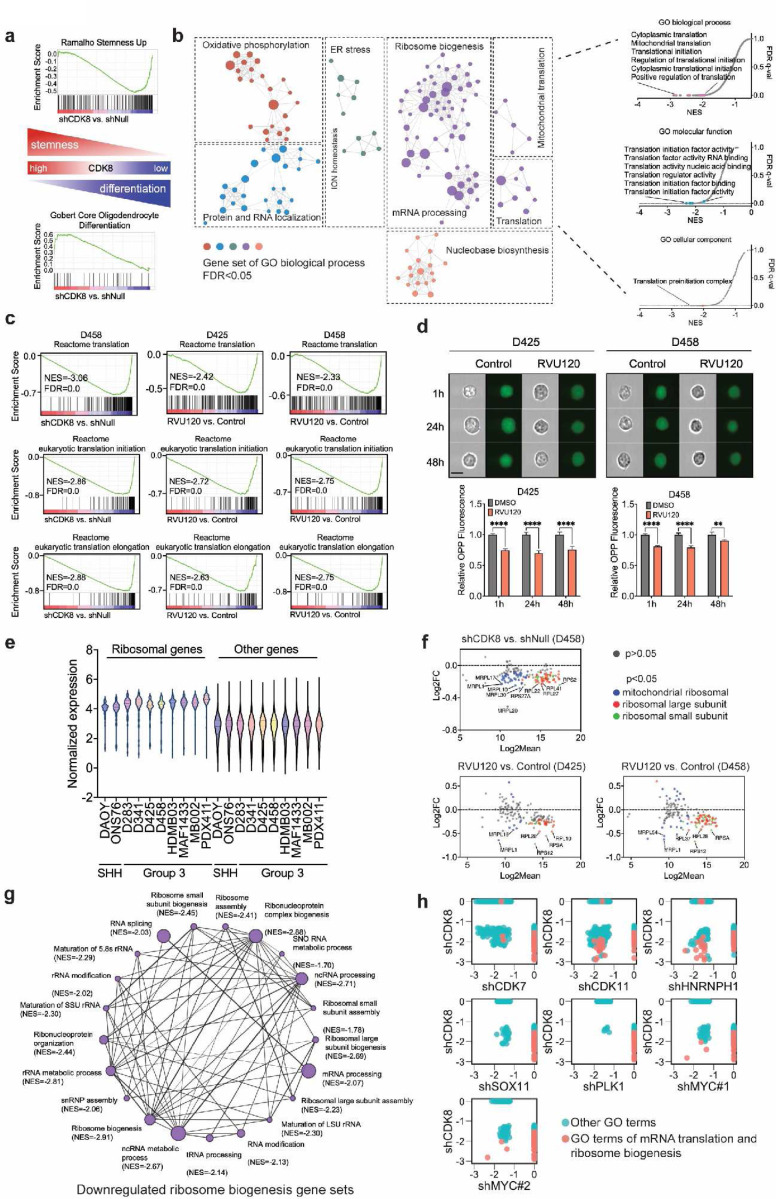
CDK8 depletion leads to repression of protein synthesis. **a.** GSEA showing the depletion of CDK8 reduced stemness gene sets and promoted differentiation gene sets in MB cells. **b.** The alterations in gene sets in D458 cells transfected with shCDK8 compared to the control. **c.** GSEA showed a downregulation of Reactome pathways associated with mRNA translation following genetic knockdown or pharmacological inhibition of CDK8 in MB cells. **d.** OPP assay for IC50 in RVU120-treated MB cells was compared with control cells, as determined by flow cytometry using FlowSight. Scale bar, 20 μm. **e.** The normalized expression of ribosomal genes was compared to that of all other genes in MB cells. **f.** RNA-Seq analysis demonstrated alterations in the expression of mitochondrial and cytoplasmic ribosomal genes. **g.** The GSEA network revealed downregulation of gene sets associated with ribosome biogenesis in shCDK8 D458 cells compared to shNull D458 cells. The plot was generated using Cytoscape, where each node represents the gene counts within specific gene sets and each line represents shared genes. **h.** GSEA indicated alterations in GO biological process gene sets (FDR < 0.05) following the knockdown of CDK8, MYC, CDK7, CDK11, HNRNPH1, SOX11, or PLK1.

To understand the mechanisms underlying CDK8 regulation, we performed RNA-Seq of MB cells after genetic knockdown or pharmacological inhibition of CDK8. CDK8 depletion altered the hallmark features of MB, including neuronal differentiation, stemness, and photoreceptor cell maintenance (Fig. S4a and Fig. 3a). Notably, many gene sets of gene ontology (GO) terms related to mRNA translation were significantly decreased (Fig. 3b). Chemical inhibition of CDK8 with RVU120 resulted in the suppression of mRNA translation, consistent with genetic depletion, further confirming the specific inhibition of CDK8 by RVU120 (Fig. 3c). To examine the functional role of CDK8 in protein synthesis, we performed an O-propargyl-puromycin (OPP) assay, which involves the introduction of a modified puromycin analog into cells, using click chemistry to visualize and quantify the rates of protein synthesis. Treatment with RVU120 led to a decrease in the OPP signal, from 1h to 48h post-treatment, demonstrating the role of CDK8 in regulating protein synthesis (Fig. 3d).

Ribosome biogenesis, which involves the coordinated assembly of ribosomal RNA (rRNA) and ribosomal proteins (RPs), plays a crucial role in regulating mRNA translation by producing functional ribosomes^10^. In MYC-driven cancer cells, including G3-MB cells, ribosomal genes typically demonstrated higher expression levels than other genes (Fig. 3e). Upon CDK8 depletion, multiple cytoplasmic and mitochondrial ribosomal genes were downregulated, leading to significant repression of gene sets associated with ribosomal biogenesis, such as ribosome assembly, ribonucleoprotein complex biogenesis, rRNA maturation, and rRNA modification (Fig. 3f,g and Fig. S4b).

MYC plays a pivotal role in regulating mRNA translation and is a primary driver of ribosome biogenesis^10^. To examine whether the alterations in ribosomal genes were due to cell death or the loss of MYC, we examined gene set alterations following knockdown of MYC or other related genes (PLK1, CDK7, CDK9, SOX11, and HNRNPH1), all of which are known to suppress MB growth and affect MYC expression^31,32,40,41^. Interestingly, MYC knockdown resulted in fewer downregulated gene sets associated with mRNA translation and ribosome biogenesis compared to the knockdown of CDK8. Remarkably, CDK8 depletion resulted in the largest number of downregulated gene sets associated with mRNA translation and ribosome biogenesis (Fig. 3h). These findings indicate a critical role of CDK8 in regulating protein synthesis in MYC-driven MB.

### CDK8 depletion leads to repression of ribosome biogenesis

**Fig. 4:**
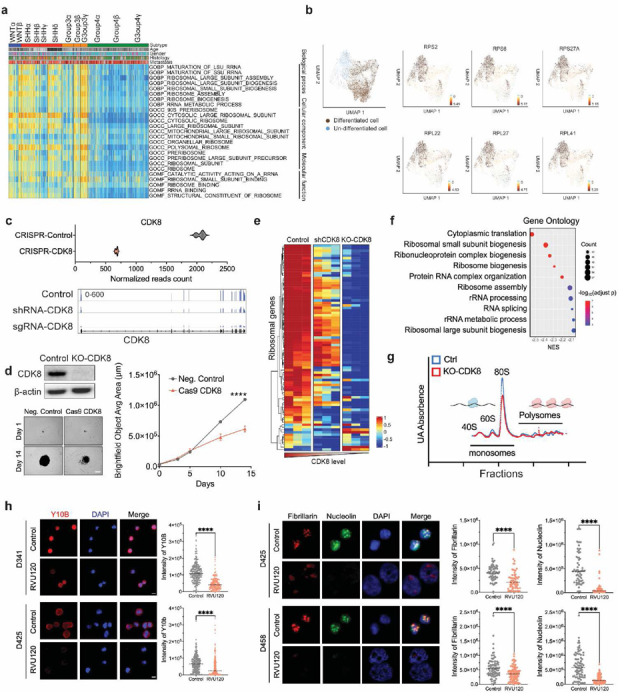
CDK8 depletion leads to repression of ribosome biogenesis. **a.** Gene set variation analysis of patient samples (n=763) revealed that the MYC-overexpressing subtypes Group3β and 3γ were enriched with gene sets related to ribosome biogenesis. **b.** Single-cell RNA-Seq analysis using MB patient samples demonstrates differentiated cells and undifferentiated cell populations (left). Representative expression of ribosomal genes is presented (right). **c.** CRISPR knockout of CDK8 in D458 cells. RNA-seq analysis shows the expression of CDK8 in knockout cells compared to control cells. IGV displays the short reads mapping to CDK8 exons in shRNA-CDK8 transfected, sgRNA-CDK8 transfected, and control cells. **d.** Immunoblot analysis of CDK8 in control and sgRNA-CDK8 transfected D458 cells. Images of neurosphere size in CDK8 knockout and control cells are shown. Scale bar, 200 μm. Knockout of CDK8 decreases the proliferation of D458 cells. **e.** RNA-Seq analysis showing the expression of cytosolic ribosomal genes in shRNA-CDK8 transfected, sgRNA-CDK8 transfected, and control D458 cells. **f.** GSEA indicates the top 10 GO biological process gene sets in knockout CDK8 D458 cells compared to control D458 cells. **g.** Polysome profiling of lysates isolated from CDK8 knockout D458 cells and control cells shows that KO-CDK8 alters the assembly of 80 monosomes. **h.** Immunofluorescence of Y10B (red, anti-ribosomal RNA) and DAPI (blue) at 40X. MB cells were treated with the IC50 of RVU120 for 48 h. Scale bar, 10 μm. **i.** Immunofluorescence of Fibrillarin (red), Nucleolin (green) and DAPI (blue) at 40X magnification. D425 and D458 cells were treated with IC50 RVU120 for 48 h. Mean ± SD. Scale bar, 10 μm. Statistical analysis: Mann-Whitney Wilcoxon test.

Hyperactive ribosome biogenesis is a feature of MB, particularly in MYC-overexpressing Group 3 MB (3β and 3γ) (Fig. 4a). Emerging evidence has shown that dysregulated ribosome biogenesis may affect cancer stem cell differentiation pathways, impacting tumor progression and therapeutic responses^42,43^. Our single-cell RNA-Seq analysis of patient samples revealed a large population of undifferentiated progenitor-like cells exhibiting high expression of ribosomal genes in G3-MB (Fig. 4b). This pattern was also observed in Group 3 MB murine models, providing a compelling rationale for considering ribosome biogenesis as a potential target for G3-MB (Fig. S5a-c).

To further investigate the role of CDK8 in ribosome biogenesis, we employed CRISPR sgRNA to achieve targeted knockout of CDK8, which resulted in a significant reduction in neurosphere growth and proliferation (Fig. 4c, d). Importantly, the loss of CDK8 led to a marked decrease in the expression of ribosomal genes compared to that in control and shRNA-transfected MB cells, indicating the role of CDK8 in regulating the transcription levels of ribosomal proteins (Fig. 4e). These gene-level alterations are associated with multiple pathways that are involved in translation, rRNA processing, and ribosome biogenesis. Notably, all top 10 gene sets identified in gene ontology biological processes were related to ribosome biogenesis and mRNA translation (Fig. 4f). Moreover, polysome profiling revealed a significant decrease in the 80S monomer peak following the loss of CDK8, indicating that CDK8 mediates changes in ribosomal activity in MYC-MB cells and is essential for continued ribosomal subunit assembly and overall protein synthesis (Fig. 4g). Furthermore, upon RVU120 treatment, we observed a reduction in 5.8S rRNA levels, as indicated by Y10b immunostaining (Fig. 4h). In agreement with these findings, we observed decreased levels of the ribosome biogenesis-associated proteins nucleolin (Ncl) and rRNA methyltransferase fibrillarin (Fbl)^44,45^ following RVU120 treatment, providing additional evidence to support the impact of CDK8 on ribosome biogenesis (Fig. 4i).

### CDK8 transcriptionally regulates the expression of ribosomal genes

**Fig. 5:**
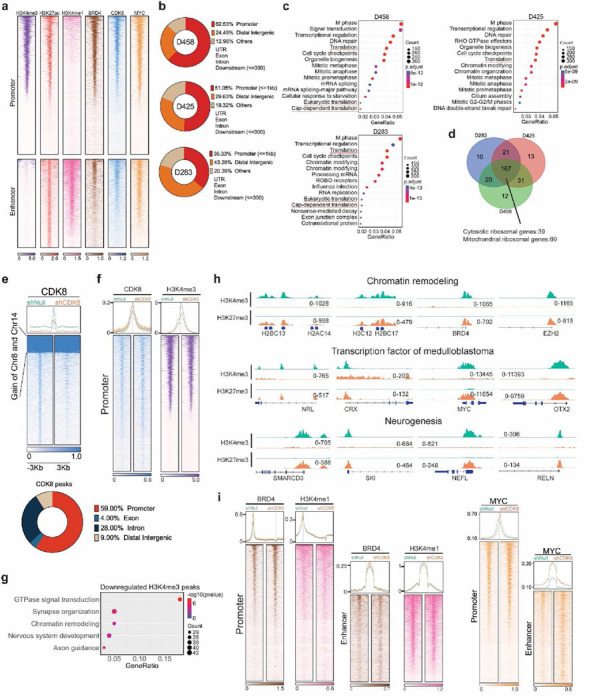
Chromatin binding profiles of CDK8 in MB cells. **a.** Heatmaps showing CUT&RUN signals of CDK8, H3K4me3, H3K4me1, H3K27ac, BRD4, and MYC in D458 MB cells. The signals were displayed within a region spanning ± 3kb around the transcription start site (TSS). **b.** Pie chart showing CDK8 peaks are localized at promoter and enhancer. **c.** Pathway enrichment analysis of CDK8 binding genes inferred from CUT&RUN. Translation pathways are enriched in MB cell lines. **d.** Venn-diagram showing overlapping of CDK8 binding genes associated with mRNA translation pathways. **e.** Heatmaps displaying genome-wide binding CUT&RUN signals of CDK8 in CDK8 knockdown D458 cells compared to control cells. The signals are displayed within a region spanning ± 3kb around the transcription start site (TSS). **f.** Heatmaps displaying CUT&RUN signals of CDK8 and H3K4me3 in D458 cells with CDK8 knockdown compared to control cells at promoter regions. **g.** Pathway enrichment analysis showing the top pathways associated with the loss of H3K4me3 peaks. **h.** Representative examples of genes with H3K4me3 and H3K27me3 peaks in chromatin remodeling, transcription factors, and neurogenesis pathways following CDK8 knockdown. **i.** Heatmaps showing CUT&RUN signals of BRD4, H3K4me1, and MYC in D458 MB cells following CDK8 knockdown.

CDK8 is a crucial component of the Mediator complex, a multi-protein assembly that plays a vital role in the transcriptional regulation of gene expression^18^. To determine whether CDK8 functions as a transcriptional activator affecting ribosomal genes, we performed a genome-wide analysis to map the occupancy of CDK8 and key histone markers using CUT&RUN in three G3-MB cell lines. CDK8 binding peaks were identified in both the promoter and enhancer regions (Fig. 5a,b). We obtained gene annotations for CDK8 binding peaks and performed functional enrichment analysis to identify the predominant biological themes among these genes. We found that pathways associated with mRNA translation were enriched in all three MB cell lines (Fig. 5c). Further analysis revealed that the predominant genes within these pathways were cytosolic and mitochondrial ribosomal genes, suggesting that CDK8 regulates the transcription of ribosomal genes (Fig. 5d).

Upon CDK8 knockdown, we found a significant decrease in its genome-wide occupancy, predominantly in promoter regions, affecting genes associated with chromatin remodeling and mRNA translation pathways, indicating a role for CDK8 in the transcriptional regulation of mRNA translation (Fig. 5e and Fig. S6a). Next, we assessed the occupancy of typical histone markers (H3K4me3, BRD4, H3K27me3, and H3K4me1). CDK8 depletion led to a significant loss of chromatin occupancy, particularly characterized by reductions in H3K4me3 at promoter regions, which are essential for gene activation and the initiation of transcription (Fig. 5f and Fig. S6b), as well as a slight decrease in H3K27me3 at the promoters (Fig. S6c). These transcriptional alterations are associated with chromatin remodeling, nervous system development, and axon guidance pathways, resulting in changes to the chromatin landscape of transcription factors and neurogenesis in MB (Fig. 5g, h). Interestingly, depletion of CDK8 led to an increased in CDK8, BRD4, and MYC signals at promoters or enhancers, suggesting that RNA Polymerase Pol II may experience promoter-proximal pausing following CDK8 depletion (Fig. 5i).

**Fig. 6:**
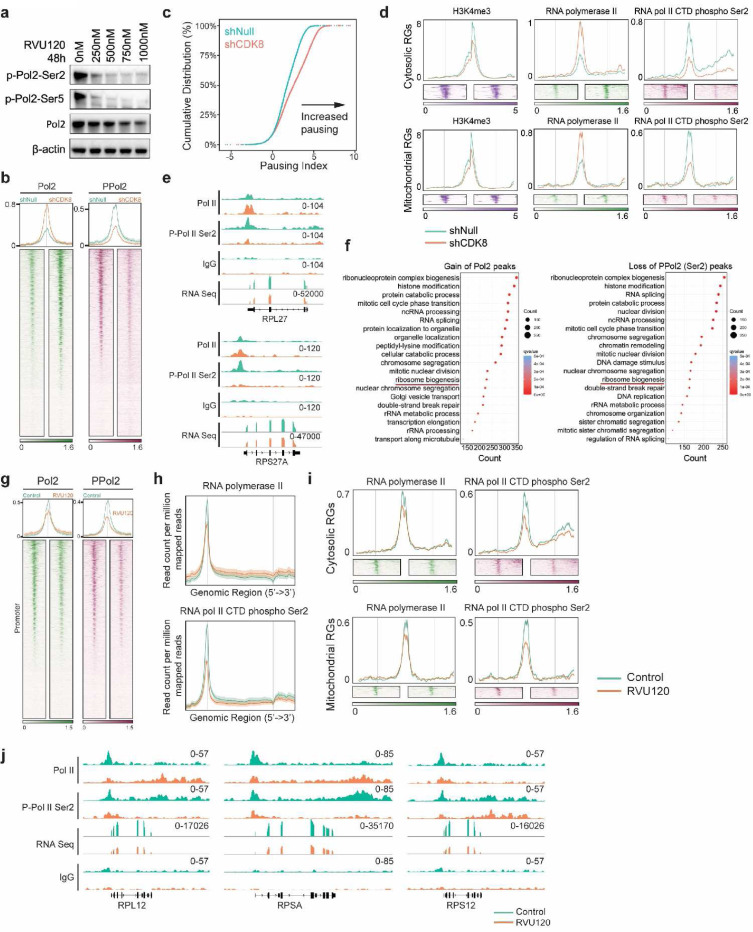
CDK8 transcriptionally regulates the expression of ribosomal genes. **a.** Immunoblot showing the levels of Pol II and phospho-Pol II in D458 cells following treatment with RVU120. **b.** Heatmaps showing CUT&RUN signals of Pol II and phospho-Pol II in D458 cells with CDK8 knockdown compared to control cells at promoter regions. **c.** Empirical cumulative distribution function (ECDF) plot shows significant increase in promoter-proximal pausing following CDK8 knockdown. **d.** Average distribution and heatmaps of H3K4me3, Pol II, and phospho-Pol II signals on ribosomal genes. **e.** Representative examples of Pol II and phospho-Pol II binding sites on ribosomal genes observed following CDK8 knockdown. **f.** Enrichment analysis identifies mRNA translation pathways are enriched among genes with an increase in Pol II peaks or a decrease in phospho-Pol II following CDK8 knockdown. **g.** Heatmaps showing CUT&RUN signals of RNA Pol II and phospho-RNA Pol II in D458 MB cells treated with IC50 RVU120 for 48 hours. **h.** Average distribution of RNA Pol II and phospho-RNA Pol II peaks showing the alteration of RNA Pol II and phospho-RNA Pol II signals across the gene body following the treatment of RVU120. **i.** Average distribution and heatmaps of RNA Pol II and phospho-RNA Pol II signals on cytosolic and mitochondrial ribosomal genes following the treatment of RVU120. **j.** Representative examples of RNA Pol II and phospho-RNA Pol II binding sites on ribosomal genes observed following the treatment of RVU120.

Therefore, we examined RNA Polymerase II and the phosphorylation of the carboxy-terminal domain (CTD) of RNA Polymerase II in MB cells. Inhibition of CDK8 with RVU120 reduced the phosphorylation levels of CTD (Fig. 6a). Knockdown of CDK8 leads to RNA Pol II predominantly pausing at the promoter regions, while the decrease in phosphorylated Pol II extends from the 5’ to the 3’ end across the gene body (Fig. 6b,c and Fig. S6d). Among the peaks showing at least a 1.5-fold change following CDK8 knockdown, we observed a greater than five-fold increase in RNA Pol II-binding sites and a three-fold decrease in phospho-Pol II-binding sites (Fig. S6e), suggesting that CDK8 regulates the phosphorylation of Pol II, thereby affecting the regulation and efficiency of gene expression. Similar chromatin alterations in Pol II and phospho-Pol II were observed in both cytosolic and mitochondrial ribosomal genes following CDK8 knockdown (Fig. 5d). These chromatin changes were associated with ribosomal gene expression, as evidenced by the overlap peak track of CUT&RUN and RNA-seq (Fig. 5e). The differential alterations in Pol II and phospho-Pol II peaks significantly contributed to various pathways associated with mRNA translation, including rRNA metabolic processes and ribosome biogenesis (Fig. 5f). Furthermore, RVU120 inhibited CDK8 activity more effectively than knockdown CDK8, leading to decreased binding of Pol II to promoters and reduced occupancy of phospho-Pol II, supporting the finding that CDK8 modulates ribosomal gene expression (Fig. 6g-j).

### CDK8 regulates mTOR signaling in MYC-driven medulloblastoma

**Fig. 7:**
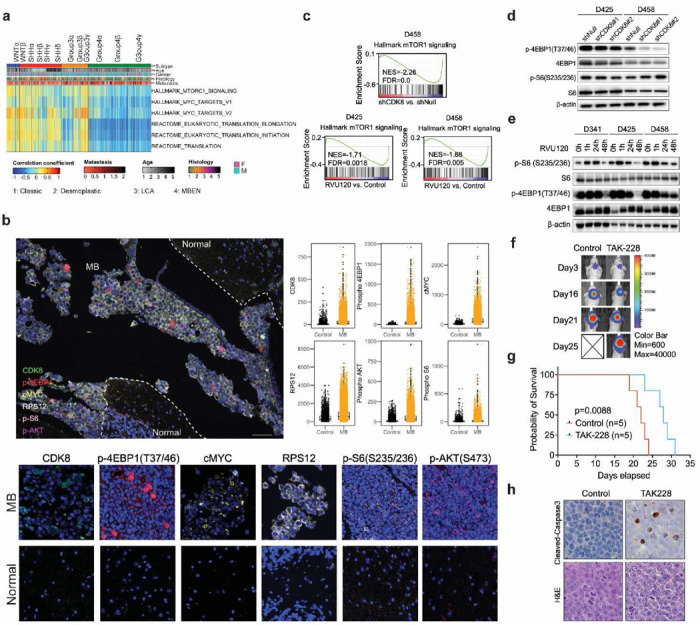
CDK8 regulates mTOR signaling in MYC-driven medulloblastoma. **a.** Gene set variation analysis of patient samples (n=763) revealed that the MYC-overexpressing subtypes Group3β and 3γ were enriched with gene sets of MYC and mTOR signaling. **b.** Multiplex IHC on G3-MB patient samples using CDK8, p-4EBP1, c-MYC, RPS12, p-S6, and p-AKT antibodies. p<0.05 in all biomarker groups. Scale bar, 100 μm. Statistical analysis: unpaired t-test. **c.** GSEA plots of representative gene sets involved in mTOR signaling following CDK8 depletion. Normalized enrichment score (NES) and false discovery rate (FDR) are indicated. **d.** Immunoblot showing the levels of p-4EBP1 and p-S6 following CDK8 knockdown. **e.** Immunoblot showing the levels of p-4EBP1 and p-S6 upon treatment with RVU120. **f.** Representative bioluminescence images of mice treated with TAK-228 (1mg/kg, daily, oral gavage) compared with those of the control cohort. **g.** Kaplan–Meier survival curves for animals treated with control or TAK-228. Statistical analysis: Log-rank test. **h.** IHC analyses of cleaved caspase 3 in xenografts mice. Three mice from each group were sacrificed 18 days after tumor implantation. Original magnification, ×40.

Aberrant protein synthesis is a common characteristic of MYC-driven cancers^46,47^. Mammalian target of rapamycin (mTOR) plays a key role in protein synthesis by regulating translational initiation, elongation, and ribosome biogenesis. Previous studies suggest that targeting mTOR could be a potential therapeutic strategy for SHH MB^48,49^. To explore the significance of mTOR signaling in MYC-driven MB, we performed gene set variation analysis (GSVA) on gene expression data from 763 MB patient samples^50^. Our analysis revealed hyperactive mTORC1 signaling, mRNA translation, and MYC signaling in G3-MB cells (Fig. 7a). Subsequently, multiplex IHC was performed on G3-MB patient samples stained for CDK8, p-4EBP (T37/46), p-S6 (S235/236), p-AKT (S473), c-MYC, and RPS12. Consistent with our gene-level findings, the staining intensity of all these protein markers was significantly higher in G3-MB than in non-tumor control regions, suggesting that targeting protein synthesis could be a potential therapeutic strategy for G3-MB (Fig. 7b and Fig. S7a,b).

GSEA of RNA-Seq data from genetic knockdown or pharmacological inhibition of CDK8 demonstrated significant downregulation of gene sets associated with mTOR signaling (Fig. 7c). To determine the effect of CDK8 on mTOR signaling, we assessed two major substrates of mTORC1: S6K1 and 4EBP1. Upon genetic knockdown of CDK8, MB cells showed decreased phospho-4EBP1 but not phospho-S6 (Fig. 7d). Time-dependent treatment with RVU120 decreased phosphorylation of both markers (Fig. 7e). Next, we evaluated the efficacy of mTOR inhibition using an MB xenograft model. Using the second-generation mTOR inhibitor TAK-228, known for its ability to penetrate the blood-brain barrier, we observed a increase in survival and enhanced apoptosis in the treated cohort compared to the control group, indicating that targeting mTOR could be a therapeutic approach for MYC-driven MB (Fig. 7f-h). These findings suggest that concurrent modulation of the CDK8 and mTOR pathways could potentially synergize to enhance therapeutic outcomes in MYC-driven MB.

### Synergistic targeting of CDK8 and mTOR in MYC-Driven medulloblastoma

**Fig. 8:**
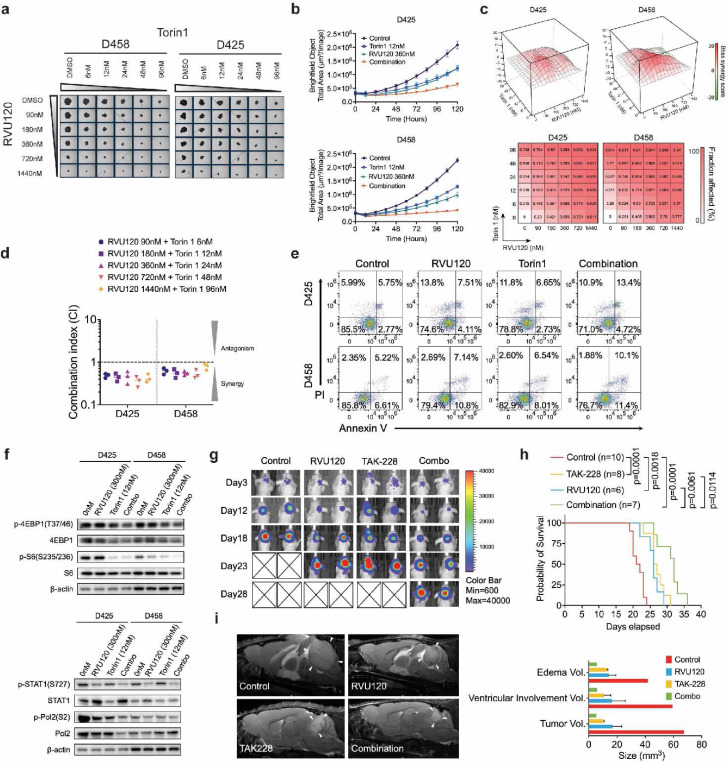
Synergistic targeting of CDK8 and mTOR in MYC-Driven medulloblastoma. **a.** Dose-dependent assay of the combined treatment with RVU120 and Torin1 on Day 5. **b.** Real-time proliferation assay quantifying the combined treatment with RVU120 and Torin1. **c.** Heatmap representation of the Fraction Affected and the Bliss interaction index across the five-point dose range of RVU120 and Torin1. Mean values of triple biological experiments are shown. **d.** The combination index of RVU120 and Torin1 using chou-talalay method. The mean combination index was determined from three independent experiment. **e.** Apoptosis assay following combined treatment with RVU120 and Torin1. MB cells were treated for 48 h before staining with PI and Annexin V. **f.** Effects of the combination of RVU120 and Torin1 on protein synthesis markers, phospho-Pol2 and phospho-STAT1, in MB cells after 48 h of treatment. **g.** The nude mice injected with D458 cells were treated with vehicle, RVU120 (40 mg/kg), TAK-228 (1 mg/kg), or their combination. **h.** Kaplan-Meier survival curve of D458 xenograft mice. Statistical analysis: log-rank (Mantel-Cox) test. **i.** Representative Sagittal T2-weighted turboRARE MRI of D458 xenografted mice at 22 days. White arrows indicate tumors. MRI volumetric analysis is shown.

Given the similar impact of mTOR and CDK8 inhibitors on the suppression of protein synthesis in MB cells, we examined whether simultaneous inhibition of CDK8 and mTOR could synergistically impede the growth of MB cells. CDK8 knockdown cells showed reduced sensitivity to Torin1, an ATP-competitive inhibitor that blocks mTORC1 and mTORC2, as demonstrated by the lower IC50 compared to control cells (Fig. S8a). Next, we performed a combination treatment study using increasing doses of RVU120 and Torin 1 on MB cells. Dual inhibition resulted in a significant synergistic effect on the lethality and proliferation of MB cells (Fig. 8a,b). Subsequent evaluation using the Chou-Talalay method and Bliss synergy model confirmed this synergistic effect (Fig. 8c,d and Fig. S8b). Flow cytometry analysis revealed that combination treatment enhanced the apoptosis of MB cells (Fig. 8e and Fig. S8c). Consistent with these results, dual inhibition significantly decreased the levels of p-4EBP1 and p-S6 and reduced p-STAT1 and phospho-Pol II levels, further emphasizing the role of CDK8 in regulating both protein synthesis and chromatin dynamics (Fig. 8f).

To explore synthetic lethality *in vivo*, we assessed the efficacy of RVU120 and TAK-228 administered individually or in combination in a D458 xenograft mouse model. Initially, the IVIS signals indicated similar tumor sizes in all groups. Tumor growth notably decelerated in the treated mice, particularly in the cohort that received the combination treatment (Fig. 8g and Fig. S8d). The RVU120 and combination-treated groups showed decreased weight loss, possibly because CDK8 is necessary for intrinsic growth and differentiation of intestinal epithelial cells (Fig. S8e). Mice receiving combination treatment showed the most effective therapeutic outcomes, characterized by prolonged overall survival and reduced tumor burden, as determined by MRI (Fig. 8h, i). Additionally, hematological analyses conducted prior to euthanizing the mice revealed that administration in each group did not induce notable acute hematological toxicity, as evidenced by the stable white blood cells, neutrophils, lymphocytes and other hematological parameters (Fig. S8f). Taken together, these studies establish the therapeutic efficacy of combination treatment with mTOR and CDK8 inhibitors *in vivo* and *in vitro*, opening an alternate path for biologically based therapeutic trials in MYC-driven MB.

## DISCUSSION

Medulloblastoma is the most common and lethal pediatric brain tumor^1,51–53^. Therefore, it is crucial to identify disease vulnerabilities and develop therapies that target specific mechanisms. Here, we identified MYC-driven medulloblastoma as one of the most significantly affected cancer types following CDK8 depletion, demonstrating the essential role of CDK8 in driving medulloblastoma growth. Our findings revealed a previously unrecognized role of CDK8 in collaborating with MYC to regulate protein synthesis, indicating its potential vulnerability in MYC-driven MB. This expands on previous studies that identified a link between CDK8 and MYC, providing a new mechanism by which CDK8 may facilitate MYC driven tumorigenesis^22,54,55^.

In G3-MBs, approximately 17% of Group 3 MB cases demonstrate high-level MYC amplification, a defining characteristic contributing to widespread treatment failure in children diagnosed with MYC-amplified MB despite current therapies^56^. MYC, which functions as a pleiotropic transcription factor, promotes the proliferation of neural progenitor cells in malignant stem cells by modulating overall gene expression and regulating critical cellular processes^57^. Although MYC can drive cerebellar stem cell proliferation *in vitro*, it is insufficient to maintain long-term growth in animal models. A previous study revealed that cerebellar stem cells require both MYC overexpression and mutant Trp53 to generate aggressive MB upon orthotopic transplantation^58^. Similar studies have demonstrated that the combination of MYC with GFI1 or MYC with SOX2 leads to rapid formation of highly aggressive cerebellar tumors using stem cells or astrocyte progenitors^59,60^. Given the significant role of CDK8 in G3-MB identified in our study, it is likely to collaborate with MYC to promote a stem cell-like state and hinder cell differentiation. It will be of great interest to determine in future studies whether CDK8 and MYC overexpression in cerebellar stem cells is sufficient to drive tumorigenesis and form G3-MB tumors.

Dysregulation of protein synthesis is a common characteristic of MYC-driven cancers and is marked by increased Pol I-mediated ribosomal rDNA transcription and mTOR/eIF4E-driven mRNA translation^12,16^. Previous studies have demonstrated the robust efficacy of PI3K/mTOR inhibitors in inhibiting the growth of MB cells derived from MYC+DNp53 transfected stem cells, both *in vitro* and *in vivo*^61^. Our findings demonstrate that G3-MB exhibits a dysregulated protein synthesis profile, predominantly comprising undifferentiated progenitor-like cells with significantly elevated expression of ribosomal genes, indicating that protein synthesis is a potential target for treatment. CDK8 depletion remarkably repressed pathways associated with ribosome biogenesis and mRNA translation. Subsequently, we investigated the mechanisms through which CDK8 regulates these cellular activities. As a dissociable part of the mediator complex, CDK8 inhibition results in decreased phosphorylation of RNA Pol II, consequently affecting the targeted suppression of gene expression, specifically of genes linked to ribosomal function. A previous study established a correlation between CDK8 and the mTOR pathway in acute lymphoblastic leukemia, suggesting that CDK8 regulates protein synthesis not only within a subset of MB but also in other types of cancer^62^. Another potential mechanism by which CDK8 affects protein synthesis is its impact on mTOR signaling, which may be mediated through the modulation of STAT1 activity^63^.

Despite the importance of CDK8 in regulating protein synthesis in MB, the mechanism by which dysregulation of protein synthesis contributes to cancer development and progression remains unclear. One possibility is that dysregulation of translation promotes cell growth, proliferation, and metastasis^64^. This is supported by the observation that cancer cells frequently develop a strong addiction to protein synthesis to adapt to different microenvironments, providing a vulnerability that can be effectively targeted by inhibiting protein synthesis in these cancer types^65^. Another possibility is that changes in translational dysregulation affect specific molecular or cellular processes that contribute to cancer initiation and progression^66,67^. Studies have demonstrated that aberrant protein synthesis leads to changes in the expression of specific genes by affecting chromatin dynamics via epigenetic mechanisms^68,69^. These findings align with the recognized role of CDK8 in the Mediator complex, suggesting that CDK8 cooperates with MYC or other transcription factors to modulate transcriptional regulation, chromatin modifications, and the overall chromatin landscape, thereby impacting gene expression and crucial cellular processes essential for development, stability, and disease states such as cancer.

We demonstrated a novel therapeutic strategy for targeting MYC-driven MB using RVU120, a new specific and selective inhibitor of CDK8^24^. RVU120 exhibits sufficient pharmacological properties such as high oral bioavailability and brain penetration. A Phase 1 trial in patients with AML or high-risk MDS (RIVER51) showed good tolerability with acceptable toxicity and signs of clinical activity (NCT04021368). As of November 2023, 38 patients have been enrolled in the RIVER51 trial without any reported dose-limiting toxicities. Pharmacodynamic studies have demonstrated target engagement with significant attenuation of CDK8 downstream biomarkers in peripheral blood monocytes and leukemic cells. Concurrently, new Phase 2 studies (RIVER-52, RIVER-81, and POTAMI-61) are underway. These clinical data support our studies, and the concept for testing RVU120 in pediatric medulloblastoma is under development. In conclusion, our data suggest that the CDK8 inhibitor RVU120 is a promising agent for MYC-driven medulloblastoma therapy and provides a mechanistic basis for future research.

## Methods

### Cell lines

The medulloblastoma cell line D425 was purchased from Millipore Sigma (SCC290). D458 was purchased from Cellosaurus (CVCL_1161), D283 from ATCC (HTB-185), and D341 from ATCC (HTB-187), respectively. MB002 was provided by Dr. Martine Roussel (St. Jude Children’s Research Hospital). HDMB03 was provided by Dr. Mahapatra of (University of Nebraska). Human astrocytes were cultured in complete Astrocyte Medium (ScienCell, 1801). MAF1433 cells were isolated and cultured from the primary tumor of a patient with G3-MB. The D425 and D458 cell lines were cultured in DMEM supplemented with 10% FBS, 1% 1× penicillin/streptomycin solution, 1% 1× L-glutamine, and 1% sodium pyruvate. D283 cells were cultured in DMEM (Thermo Fisher) supplemented with 10% FBS, 1 mM sodium pyruvate, 1× penicillin/streptomycin solution (Cellgro), and 1× nonessential amino acids (Millipore Sigma). HDMB03 cells were cultured in 90% RPMI 1640, 10% FBS, and 1× penicillin/streptomycin. D341 and MB002 were cultured in neurobasal medium (Sigma, SCM003) containing 2% B-27, 1 μg/ml heparin, 2 mM L-glutamine, 1% penicillin/streptomycin, 25 ng/ml fibroblast growth factor (FGF), and 25 ng/ml epidermal growth factor (EGF). All cell lines were cultured at 37°C in 95% air and 5% CO_2_. All cell lines tested negative for Mycoplasma. Cell proliferation assays and live-cell imaging were performed using an Incucyte SX5 Live-Cell Analysis System (Sartorius).

### Transfection

shRNA vectors targeting CDK8 mRNA (#TRCN0000350344 and #TRCN0000382350) and a non-targeting shRNA (control) were purchased from the Functional Genomics Facility at the University of Colorado Anschutz Medical Campus. Transfection was performed using the Lipofectamine 3000 Transfection Reagent (Invitrogen).

### Methylcellulose assay

2000 cells/3 mL were plated in a 1:1 mixture of 2.6% methylcellulose and complete growth medium. The cells were allowed to grow for two weeks. Colonies were stained with nitrotetrazolium blue chloride (Sigma) at 1.5mg/mL in PBS for 24 h at 37°C and counted.

### Aldehyde dehydrogenase assay

ALDH activity was measured using an Aldefluor kit (Stem Cell Technologies), according to the manufacturer’s instructions. Briefly, 1 × 10^5^ cells were resuspended in 0.5 mL Aldefluor buffer, separated equally into two tubes, and 5 μl of DEAB reagent was added to one tube as a negative control. Then 1.25 μl of Aldefluor Reagent was added to each tube and mixed well. After incubation at 37°C for 45 min and centrifugation, the cells were stained with propidium iodide and analyzed using a FlowSight Imaging Flow Cytometer (EMD Millipore).

### Neurosphere assay

Medulloblastoma cells were grown for 14 days in neurosphere medium. The spheres were disassociated and replanted into 100-, 10-, and single-cell suspensions on day 14. The cells were grown for an additional 14 days with or without RVU120. The spheres were imaged using an Incucyte S3 Live Cell Imaging System (Sartorius).

### Immunofluorescence

The cells were washed and seeded onto polylysine-coated slides, and then fixed with 4% paraformaldehyde for 15 min at room temperature, permeabilized with 0.2% Triton X-100 in PBS for 15 min, and incubated in 3% BSA diluted in 0.05% Triton X-100 for 30 min at room temperature on a shaker. After blocking, the cells were incubated with the primary antibodies. The following antibodies were used: phospho-4EBP1 (Santa Cruz Biotechnology, sc-293124, 1:50), CDK8 (Santa Cruz Biotechnology, sc-13155, 1:50), CDK8 (Abcam, ab224828, 1:200), CDK8 (Invitrogen, PA5–11500, 1:200), fibrillarin (Abcam, EPR10823, 1:200), nucleolin (Abcam, EPR7952, 1:200), and ribosomal RNA antibody Y10B (Abcam, ab171119, 1:200) for 1 h at room temperature. After washing with 0.05% Triton X-100, cells were incubated with Alexa Fluor 647-or Alexa Fluor 488 conjugated secondary antibody (1:500) for 1 h at room temperature in the dark, washed with PBS, and mounted using ProLong Gold antifade reagent containing DAPI (Sigma). Images were acquired using an inverted epifluorescence microscope at × magnification of 40x.

### Western blotting

Western blotting was performed as described previously^31^. Antibodies used for western blot analysis were from the following sources: β-actin (Cell Signaling, 8457, 1:2000), CDK8 (Cell Signaling, 4101, 1:1000), 4EBP1 (Cell Signaling, 9644S, 1:1000), phospho-4EBP1 (Cell Signaling, 2855S, 1:1000), STAT1 (Cell Signaling, 9176S, 1:1000), phospho-STAT1 (Cell Signaling, 8826S, 1:1000), S6 (Cell Signaling, 2217T, 1:1000), phospho-S6 (Cell Signaling, 4858T, 1:1000), RNA Pol II (Cell Signaling, 2629S, 1:1000), and phospho-RNA Pol II-Ser2 (Cell Signaling, 13499, 1:1000).

### Compounds

The CDK8 inhibitors RVU120, Torin1 and TAK-228 were purchased from MedChemExpress, and RVU120 for animal studies was provided by Ryvu Therapeutics. The drugs were reconstituted in dimethyl sulfoxide (DMSO). An equivalent amount of DMSO at the highest concentration of the drug was used for each experiment as a vehicle control.

### Extreme limiting dilution assay

The cells were treated with the indicated concentrations of RVU120 and then seeded into 96-well ultra-low-attachment plates in neurosphere media at increasing concentrations from 1 to 250 cells/well. Cells were seeded from n = 5 wells (250 cells/well, 100 cells/well), n = 10 wells (10–50 cells/well), or n = 30 wells (1 cell/well) per condition. The cells were allowed to grow for 14 days, and the number of wells containing neurospheres was counted under a microscope.

### Protein synthesis assay

The MB cells were plated at a density of 2,000 cells/well in a 96-well plate and cultured overnight. The next day, the cells were treated with either vehicle or RVU120 for 1, 24, or 48 h. The cells were then collected and centrifuged at 400 × g and resuspended in OPP (O-propargyl-puromycin) working solution (Cayman Chemical, 601100). The mixed cells were incubated for 30 min at 37°C for OPP labeling of translated peptides. Following incubation, cells were fixed, washed, and analyzed using flow cytometry.

### Drug interaction assay

Medulloblastoma cells were plated in 96-well low-attachment plates and subjected to dose-response assessments for individual drugs, as well as various concentrations of drug combinations, with DMSO (0.1%) and media serving as controls. The growth inhibition was quantified using the CellTiter 96 AQueous Non-Radioactive Cell Proliferation Assay (Promega) and the Incucyte SX5 Live-Cell Analysis System (Sartorius). At least five independent trials were conducted to ensure reproducibility of the results. The Chou-Talalay median-effect model and the Bliss independence dose-response surface model were used to classify whether the two drugs interacted in an antagonistic, additive, or synergistic manner. For the Chou-Talalay median-effect model, CI > 1 indicated antagonism, CI = 1 demonstrated activity, and CI < 1 indicated synergistic interactions.

### Unbound Brain-to-Plasma Partition Coefficient (Kpuu)

RVU120 was given to animals as a single dose of 10 mg/kg by an intravenous (rats, due to limited bioavailability in rats) and oral (mice) administration. At predefined time points (4 hr for mice, 2 hr for rats) animals were anesthetized and blood samples were collected by heart puncture using a heparinized syringe and centrifuged at 4°C and 4,000 g for 5 min to obtain plasma. Immediately after the final blood sample was obtained, the lumbar CSF collected by a single lumbar puncture. Plasma CSF samples were stored at −20°C until use.

The quantification of RVU120 in plasma and CSF samples was performed using liquid chromatography – tandem mass spectrometry method (LC/MS/MS). Briefly, the proteins in the samples (55 μL of plasma or 10 μL of CSF) were precipitated with 200 uL acetonitrile and, centrifugated at 4°C and 10,000 g for 15 min and the supernatants were injected on LC/MS/MS. Compound was analyzed in multiple reaction monitoring (MRN) mode using a Sciex QTrap 5500 instrument (Torrance, MA, USA) equipped with Shimadzu DGU-20A5R(C) LC system (Kyoto, Japan) with Phenomenex Kinetex C18, 2.6μ 100A, 30*2.1 mm; (Torrance, CA, USA) as analytical chromatography column.

The unbound fraction of compound in murine and rat plasma was determined by equilibrium dialysis with Rapid Equilibrium Dialysis (RED) Device (Thermo Fisher Scientific, Rockford, IL, USA). Plasma samples were spiked with the test compound (1 or 5 μM) and were dialyzed versus buffer (150 mM sodium phosphate buffer). The 96-well equilibrium dialysis apparatus was maintained on a rotator (set at 100 rpm) in an incubator at 37°C for 18 h. Samples, after unifying matrix and protein precipitation, were vortexed and centrifuged for 20 minutes at 4°C at 2000 g, supernatants were transferred into the HPLC plate for LC-MS analysis.

CSF-to-plasma unbound concentration ratios (${\text{K}}_{\text{p,uu}}$) were calculated as follows:

$$K_{p,uu}=\frac{C_{CSF}}{C_{p}\times f_{p}}$$

Where ${\text{C}}_{\text{CSF}}$, ${\text{C}}_{\text{p}}$, ${\text{f}}_{\text{p}}$ represent respectively CSF concentration, plasma concentration and the unbound fraction in plasma.

### RNA-seq

RNA was isolated from cells under the indicated experimental conditions using a Qiagen miRNAeasy kit (Valencia) and measured using an Agilent Bioanalyzer (Agilent Technologies). Illumina Novaseq 6000 libraries were prepared and sequenced by Novogene (CA, USA) or the Genomics and Microarray Core Facility at the University of Colorado Anschutz Medical Campus. High-quality base calls at Q30 ≥ 80% were obtained with approximately 40 M paired paired-end reads. Sequenced 150bp pair-end reads were mapped to the human genome (GRCh38) by STAR (v2.4.0.1), read counts were calculated by R Bioconductor package GenomicAlignments (v1.18.1), and differential expression was analyzed with DESeq2 (v1.22.2) in R. Further analysis by GSEA was performed using GSEA (v2.1.0) software with 1,000 data permutations and Cytoscape (v3.10.1).

### Gene set enrichment analysis

Gene sets from MSigDB were downloaded and used to estimate biological activity. The ssGSEA algorithm in the R package GSVA (v.1.40.1) was applied to estimate signature enrichment in the bulk transcript datasets. The enrichment results of GO and pathways among differentially expressed genes were generated using the R package clusterProfiler (v.4.7.1).

### CUT&RUN

A total of 500,000 cells per reaction were harvested and captured using 10 μL of pre-activated ConA beads (EpiCypher). Beads with attached cells were incubated at room temperature for 10 min to ensure complete adsorption. Subsequently, 50 μL of cold antibody specific to the reaction was added to each sample. The antibodies used for CUT&RUN were CDK8 (Cell signaling, 4101S, 1:50), MYC (Cell Signaling, 13987S, 1:50), RNA Pol II (Cell signaling, 2629S,1:50), phospho-RNA Pol II (Cell Signaling, 13499S, 1:50), H3K4me1 (Abcam, ab8895, 1:50), H3K4me3 (EpiCypher, 13–0041K, 0.5 mg/ml), BRD4 (Cell signaling, 13440S, 1:50), H3K27ac (Active motif, 39133, 1:25), and IgG (EpiCypher, 13–0042K, 0.5 mg/ml). The cells were then incubated overnight on a nutator at 4 °C and permeabilized using a buffer containing 5% digitonin. Next, 2.5 μL/reaction pAG-MNase (Epicypher) was added to each sample. The beads were gently resuspended by vortexing or pipetting to evenly distribute the enzymes. The mixture was incubated for 10 min at room temperature. Calcium Chloride (100 mM, 1 μL/reaction) was added to the reaction, followed by a 2-hour incubation at 4 °C. After incubation, 34 μL of Stop Master Mix was added to each tube, followed by a 10-minute incubation at 37 °C. The tubes were then quick-spun and placed on a magnet for slurry separation, and the clear supernatants were transferred to 8-strip tubes for DNA purification. Libraries were prepared using the NEBNext Ultra II DNA Library Prep kit and sequenced using NovaSeq PE150.

CUT&RUN-seq reads were aligned to the reference human genome hg38 using BOWTIE (v.2.3.4.1). Aligned reads were stripped of duplicate reads using Sambamba (v.0.6.8). Peaks were called using the program MACS (v2.1.2), with the narrow peak mode using matched input controls and a q-value of 0.00001. Peaks in the blacklisted genomic regions identified by the ENCODE consortium were excluded using bedtools. For downstream analysis and visualization, bamCoverage was used to generate bigwig files and density maps were produced using IGV tools. Group 3 medulloblastoma enhancers were defined based on H3K27ac signals. Regions within 1kb of RefSeq transcription start site (TSS) locations and peaks with strong H3K4me3 signals typical of active promoters were subtracted from these signals. Annotation and visualization of the peaks were conducted using ChIPseeker (v3.18). Differentially marked genes were calculated using DiffBind and DESeq2, based on the threshold of FDR < 0.05 and fold-change ≥ 2.

### Single cell RNA-seq

Single-cell RNA sequencing data were aligned against a composite reference consisting of mm10 and hg38 genomes to delineate transcripts originating from murine and human cancer cells using the Cell Ranger toolkit (version 4.0.0). The classification of cells as either human or murine was based on a threshold of 90% genome-specific reads. Cells falling below this threshold were identified as human-mouse chimeric multiplets and excluded from further analysis. Gene-barcode count matrices obtained from scRNA-seq were processed using the Seurat package (version 4.0.3) in R. Cells with fewer than 500 or more than 8,000 genes were excluded to eliminate low-quality samples and potential doublets. Cells with over 10% reads mapped to mitochondrial genes were filtered out. Log-normalization was applied to the filtered datasets, followed by principal component analysis to reduce the dimensionality. Utilizing Seurat’s elbow plot function, the top 25 principal components were selected for UMAP plot generation. Cell clusters were discerned via k-nearest neighbor unsupervised clustering and the resolution parameter was set to 1.2. Established markers from literature were used to annotate each cluster with its corresponding biological cell type.

### Multispectral IHC

Tumor tissues were fixed in formalin and paraffin-embedded for multispectral imaging using the Vectra 3.0 Automated Quantitative Pathology Imaging System (Perkin Elmer). Four-micron sections mounted on glass slides were sequentially stained for human CDK8 (Abcam, ab224828), MYC (Abcam, ab168727), RPS12 (Abcam, ab167428), p-4EBP1-T37/46 (Abcam, ab75831), p-S6-S235/236 (Cell Signaling, 2211S), p-AKT-S473 (Leica, NCL-L-AKT-PHOS), and DAPI using a Bond RX autostainer (Leica). Slides were dewaxed (Leica), heat-treated in ER2 (epitope retrieval solution 2) antigen retrieval buffer for 20 min at 93 °C (Leica), blocked in antibody (Ab) Diluent (Perkin Elmer), incubated for 30 min with the primary antibody, 10 min with horseradish peroxidase-conjugated secondary polymer (anti-mouse/anti-rabbit, Perkin Elmer), and 10 min with horseradish peroxidase-reactive OPAL fluorescent reagents (Perkin Elmer). Slides were washed between staining steps with Bond Wash (Leica) and stripped between each round of staining by heat treatment in antigen retrieval buffer. After the final round of staining, the slides were heat-treated in ER1 antigen retrieval buffer, stained with spectral 4′,6-diamidino-2-phenylindole (Perkin Elmer), and coverslipped with ProLong Diamond mounting media (Thermo Fisher). Whole slide scans were collected using a 10× objective lens at a resolution of 1.0 μm. Approximately 30 regions of interest were selected from the tumor in areas near the tumor border or in the center of the tumor. Regions of interest were scanned for multispectral imaging with a 20× objective lens at a resolution of 0.5 μm. Multispectral images were analyzed using inForm software version 2.3 (Perkin Elmer) to unmix adjacent fluorochromes, subtract autofluorescence, segment the tissue into tumor regions and stroma, segment the cells into nuclear, cytoplasmic, and membrane compartments, and phenotype the cells according to cell marker expression.

### Animal studies

Female athymic Nude *Foxn1*^nu^ and female NOD scid (NSG, #5557) gamma mice aged 4–8 weeks were used for orthotopic xenograft studies. D458 cells were collected and resuspended in a single cell suspension of 20,000 cells/3 μl in non-FBS medium. The mice were monitored daily for tumor growth and euthanized when 15% weight loss was reached. To monitor tumor growth in D458 xenograft mice, the mice were injected intraperitoneally with 10 μl/g of 15 mg/mL D-luciferin potassium salt solution (Gold Biotechnology) and imaged using the Xenogen IVIS 200 In Vivo Imaging System (PerkinElmer). Tumor bioluminescence was analyzed using the Living Image 2.60.1 software (PerkinElmer). The mice used in this study were kept in a sterile envrioment under 12/12-h light/dark cycle, 21–23 °C and 40–60% humidity at University of Colorado, Anschutz Medial Campus, Aurora, USA.

The mice were administered a daily dose of 40 mg/kg RVU120 or 1 mg/kg TAK-228 via oral gavage. RVU120 was dissolved in water and TAK-228 was prepared by dilution in N-methyl-2-pyrrolidone (NMP) and subsequent suspension in a 15% polyvinylpyrrolidone solution for administration. In the combination treatment group, mice received RVU120 initially, followed by a 2-hour intermission before the administration of TAK-228. All mice were treated with the respective drugs 2–4 hours before sacrifice, and blood was extracted for hematological toxicity analysis.

### Study approval

All patients provided written informed consent for molecular studies of their tumors, and the study protocol was approved by the ethics committee of the University of Colorado and Children’s Hospital Colorado (COMIRBs #95–500). All animal procedures were performed in accordance with the National Research Council’s Guide for the Care and Use of Laboratory Animals and approved by the University of Colorado, Anschutz Campus Institutional Animal Care and Use Committee.

### Statistics analysis

Statistical significance was set to P < 0.05. The neurosphere sizes between knockout or knockdown of CDK8 vs. control were compared using one-way ANOVA (N=5 biologic replicates). Immunofluorescence of CDK8 were compared using Mann-Whitney Wilcoxon test. Methylcellulose assays were compared using two-way ANOVA. The neurosphere sizes in RVU120 treated MB cell lines were compared using one-way ANOVA (N =5 biologic replicates). Annexin V apoptosis assay of RVU120 treated MB cells vs. control were analyzed using student t-test. Immunofluorescence of Y10B were compared using Mann-Whitney Wilcoxon test. ALDH flow cytometry were analyzed using one-way ANOVA (N =3 biologic replicates). Multiplex IHC on G3-MB patient samples were compared using unpaired t-test. The statistical analysis of CUT&RUN peaks of CDK8, H3K4me3, RNA Pol II, phospho-RNA Pol II, and BRD4 were calculated using one-way ANOVA. Transcriptomics data were analyzed using DESeq2 with an adjusted P value threshold of 0.05. R2: The Genomics Analysis and Visualization Platform (https://hgserver1.amc.nl/cgi-bin/r2/main.cgi?open_page=login) was used to delineate the association between gene expression levels and overall survival in patient samples. For survival analysis of patient samples and xenograft mice, log-rank (Mantel-Cox) test was used. The log-rank P values, and Kaplan–Meier curves were calculated and plotted using the R package survival (v.3.2–11) and Prism GraphPad (v.10.0.2). R package survival (v.3.2–11) and Prism GraphPad (v.10.0.2) were used for the statistics.

## Supplementary Material

Supplement 1
