# Supplementary material for: Transcriptional Regulation of Protein Synthesis by Mediator Kinase Represents a Therapeutic Vulnerability in MYC-driven Medulloblastoma
Source: Res Sq. 2024 Nov 1:rs.3.rs-5329081. Preprint. [Version 1] doi: 10.21203/rs.3.rs-5329081/v1 (PMC11581124; doi:10.21203/rs.3.rs-5329081/v1)
Supplement: Supplement 1 [file NIHPPRS5329081V1-supplement-1.pdf]

Supplementary Files

This is a list of supplementary files associated with this preprint. Click to download.

- SupplementaryInformation.pdf
